# Supplementary material for: Multipole plasmons and their disappearance in few-nanometre silver nanoparticles
Source: Nat Commun. 2015 Nov 5;6:8788. doi: 10.1038/ncomms9788 (PMC4659934; doi:10.1038/ncomms9788)
Supplement: Supplementary Information — Supplementary Figures 1-14, Supplementary Notes 1-6 and Supplementary References [file ncomms9788-s1.pdf]

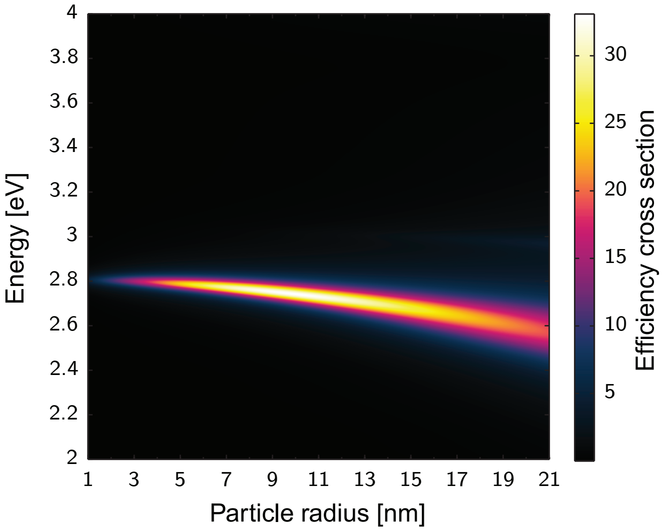

Supplementary Figure 1: Extinction efficiency cross section map. Color plot of cross-sectional efficiency as a function of energy and radius for spherical silver nanoparticles fully embedded in a medium with permittivity $\varepsilon_B = 3.3$. Due to the plane wave excitation, only the dipole mode is efficiently excited.

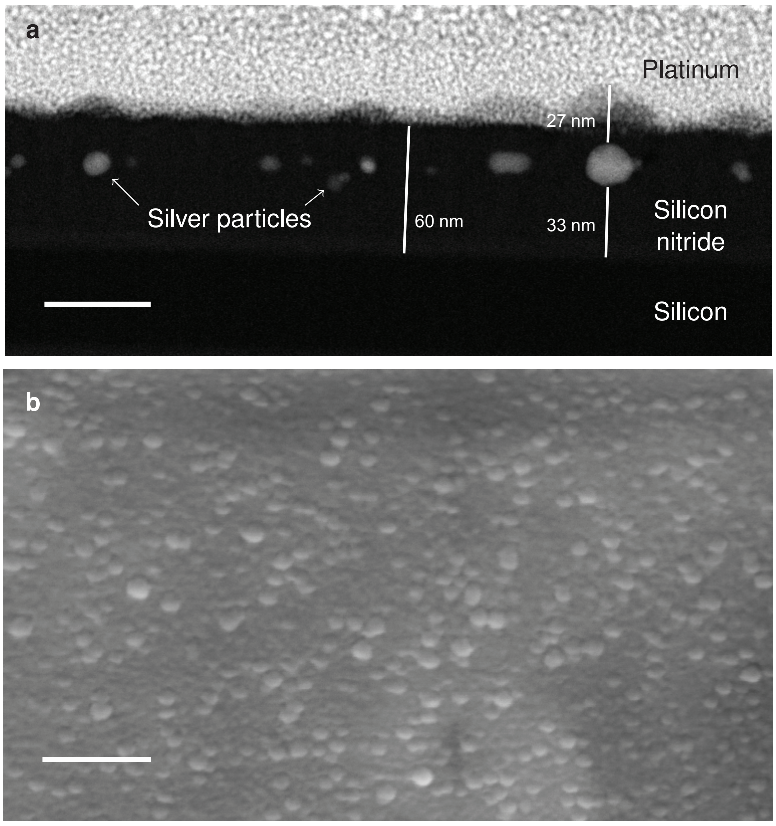

Supplementary Figure 2: Conformal coating - FIB cross section and SEM image. (**a**) STEM image of the cross section of one of our samples, prepared using FIB, with labels denoting the different materials. The platinum on top originates from the FIB lamella procedure and is not present during our EELS measurements. Scale bar is 50 nm. (**b**) Scanning electron microscope image displaying, from the top, the same sample without the platinum. Clear bumps in the surface topography due to the conformal coating of the silicon nitride are present. The sample shown in this figure has a slightly thicker silicon nitride coating (approximately 60 nm in total) than the one used for the EELS measurements in the main text (approximately 30 nm in total). Scale bar is 200 nm.

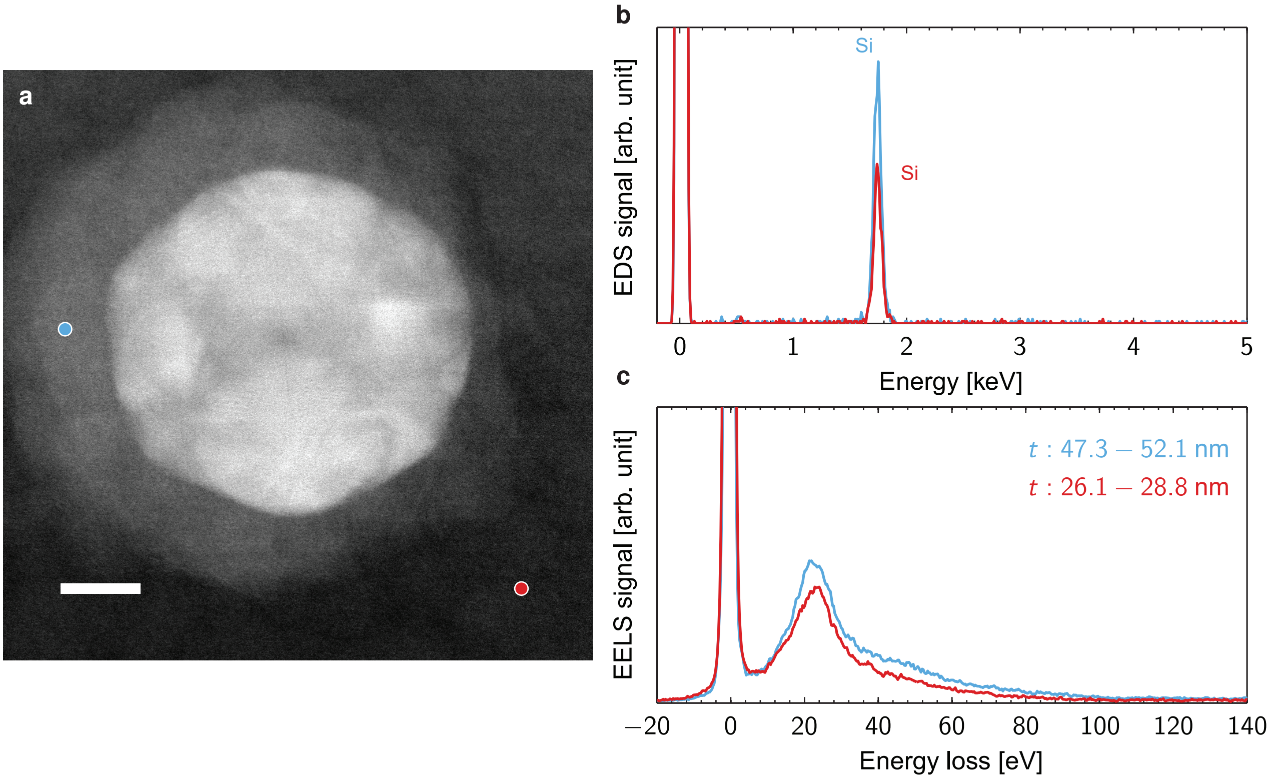

Supplementary Figure 3: Conformal coating - EDS and EELS signals. (**a**) STEM image of silver nanoparticle with radius $R = 22.1 \pm 0.7$ nm encapsulated in silicon nitride. Scale bar is 10 nm. (**b-c**) EDS and EELS signals, respectively, acquired from the impact positions shown in (**a**). The legend in (**c**) designates the position-dependent thickness t of the silicon nitride layer due to the conformal coating. The thickness intervals are extracted from the EELS data using the log-ratio method.[1]

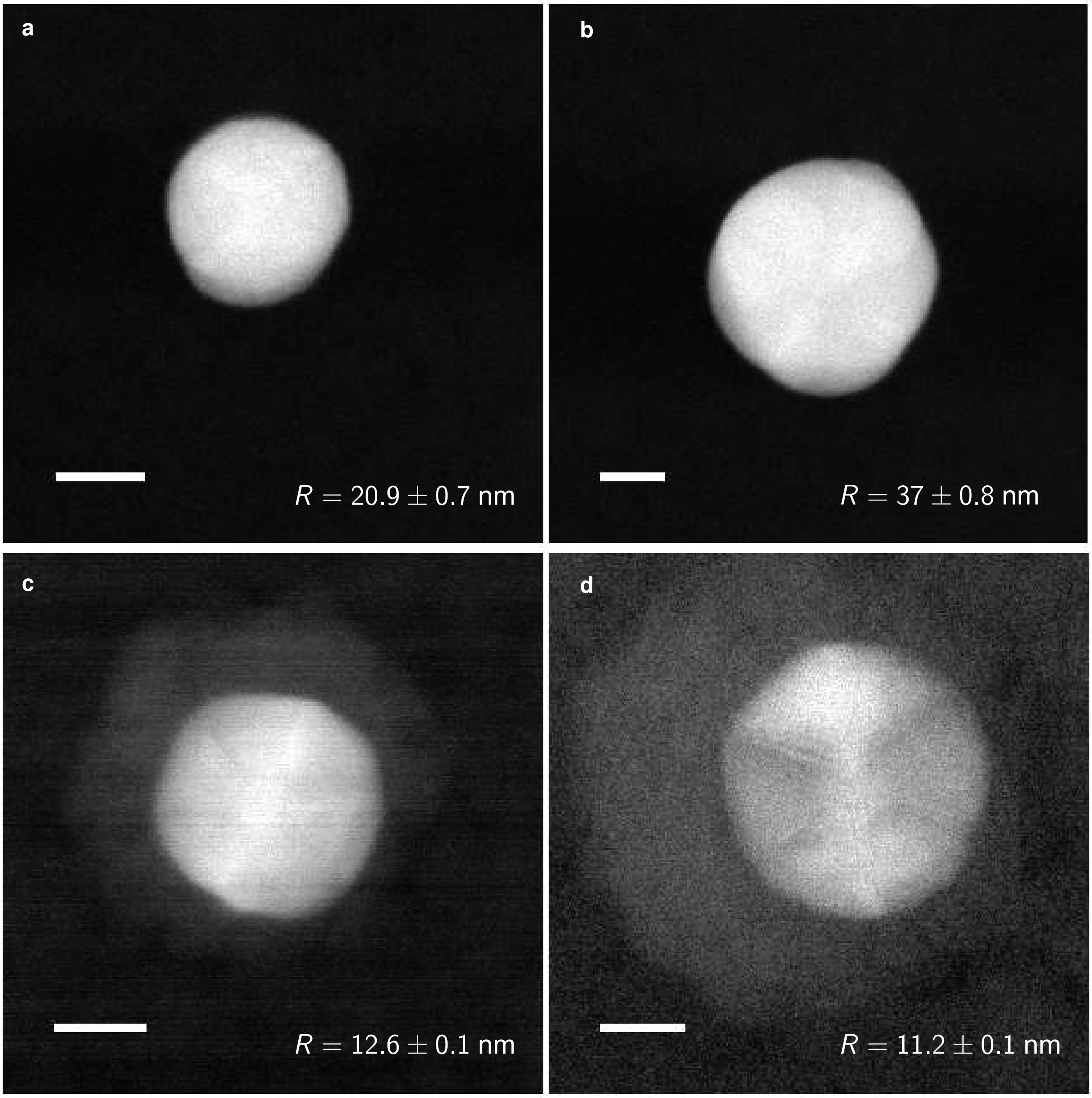

Supplementary Figure 4: Silver nanoparticles with and without silicon nitride encapsulation. STEM micrographs of (**a-b**) non-encapsulated bare silver nanoparticles (scale bars are 20 nm) and (**c-d**) encapsulated silver nanoparticles (scale bars are 10 nm). The nanoparticles are deposited on a 20 nm thick silicon nitride substrate and the encapsulating medium is also silicon nitride.

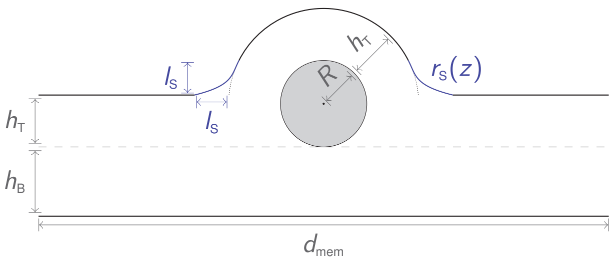

Supplementary Figure 5: BEM simulation domain. Side-view of the radially-symmetric geometry used for the BEM EELS simulations. The spherical Ag nanoparticle of radius R is conformally encapsulated by top and bottom layers of SiN_x with thickness h_T and h_B, respectively. The encapsulating layer is modelled as a circular membrane of diameter d_{mem} and height $h_T + h_B$, forming a union with a hemispherical cap centered at the center of the particle and with a radius of $R + h_T$. A smoothing profile $r_S(z)$ is applied at the region defined by the length l_S, which connects the hemispherical cap and the circular membrane.

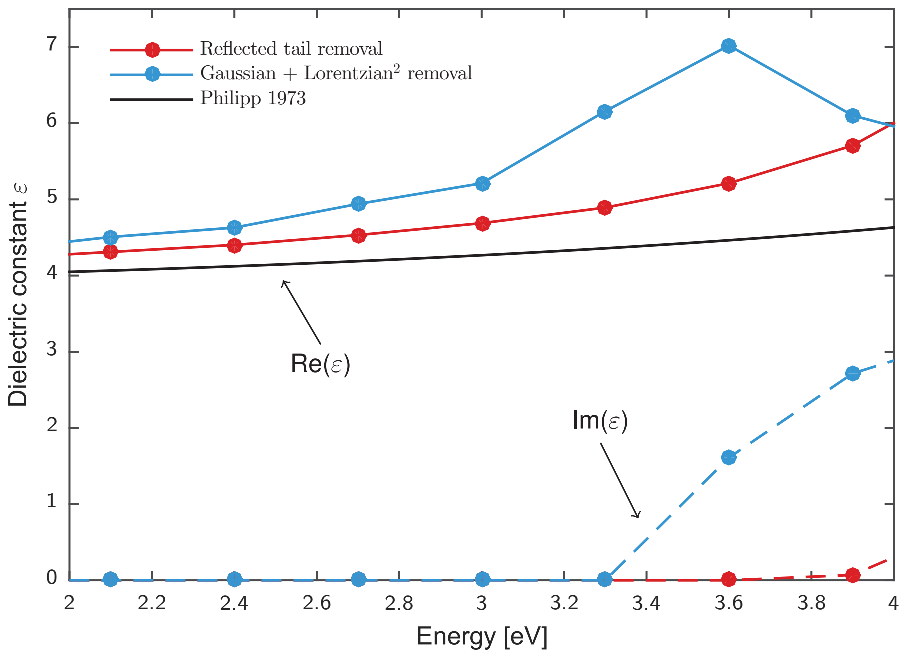

Supplementary Figure 6: Permittivity of silicon nitride. Real and imaginary parts of the dielectric constant of deposited silicon nitride coating extracted from EELS data using the Kramers–Kronig method.[1] The red curve shows the result with the reflected tail method for removal of the zero-loss peak, while for the blue curve the sum of a Gaussian and squared Lorentzian function is used to fit the zero-loss peak. The black line shows the tabulated values for bulk silicon nitride.[2] For the Kramers–Kronig method,[1] the following values are used: collection semi-angle $\theta_{\mathrm{coll}} = 16$ mrad, convergence semi-angle $\theta_{\mathrm{conv}} = 16$ mrad, and optical refractive index $n_{\mathrm{opt}} = 2$.

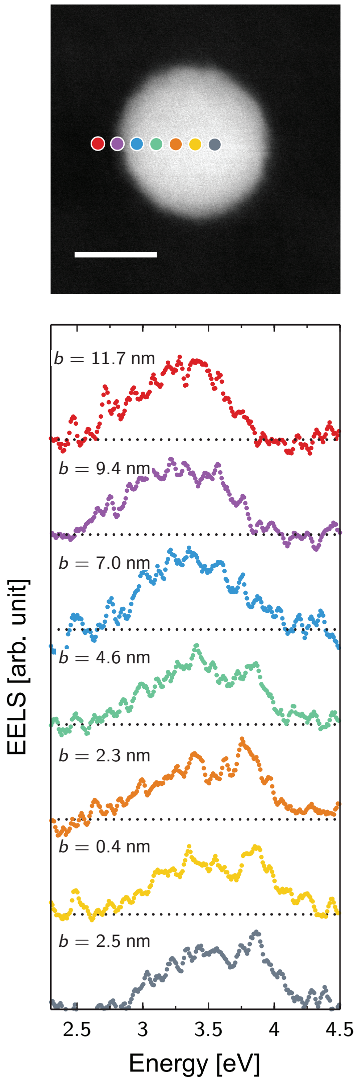

Supplementary Figure 7: EELS data of non-encapsulated silver nanoparticle. Same as the experimental part of Figure 3 in the main manuscript, but for a non-encapsulated silver nanoparticle with similar size $R = 9.1 \pm 0.2$ nm. The silver nanoparticle is situated on a 20 nm thick silicon nitride substrate. The EELS data show a single surface plasmon excitation at approximately 3.5 eV, corresponding to the dipole mode. In contrast to the encapsulated nanoparticles studied in the main manuscript, we observe no additional peak that would correspond to HO modes. Scale bar is 10 nm.

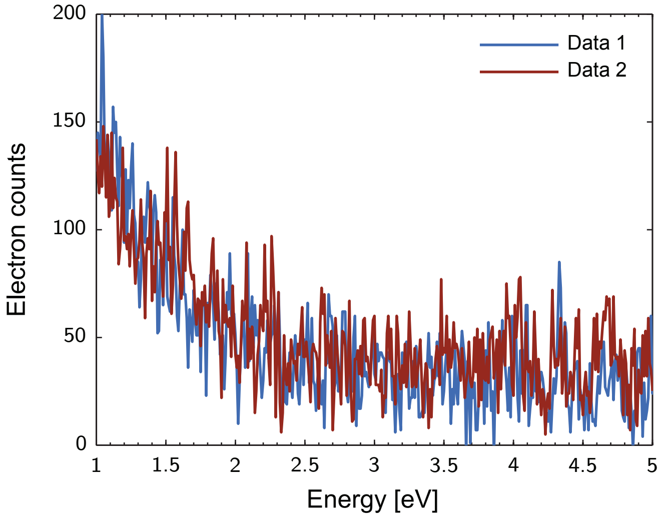

Supplementary Figure 8: EELS data of silicon nitride. Raw EELS data acquired through silicon nitride away from the silver nanoparticles. The EELS data are featureless in the $2 - 4$ eV range, confirming that the observed HO modes are a feature of the silver nanoparticles rather than the encapsulating medium. The shoulder below 2 eV is the tail of the zero-loss peak.

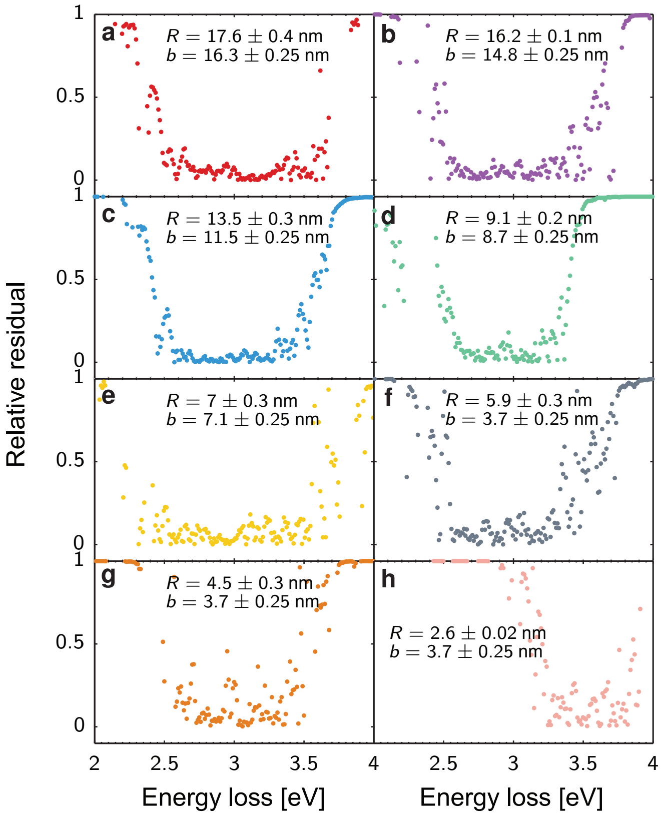

Supplementary Figure 9: Residual plot of Figure 4. (**a-h**) Absolute value of EELS data with the fit of Gaussian functions being subtracted, relative to (i.e., divided by) the EELS data (compare with Figure 4 of the main text). The good fit of the sum of two Gaussian functions in (**a-g**) (which can be inferred from the vanishing relative residual in the energy range $\sim 2.5 - 3.5$ eV) shows that the HO modes peak is accurately captured by a single (symmetric) Gaussian function. The non-negligible residual for energies above 3.5 eV is due to EELS signal from the bulk plasmon, which is not accounted for in our two-Gaussian fit.

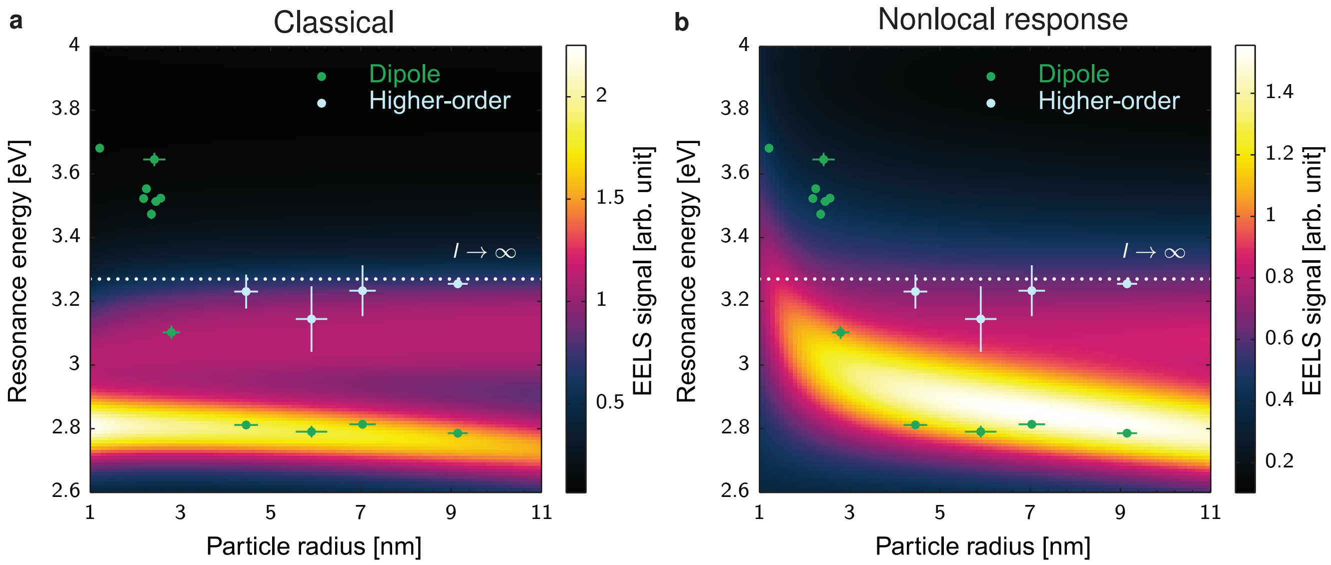

Supplementary Figure 10: Fully-embedded approximation and nonlocal response. (**a**) Color plot of simulated EELS signal for silver nanoparticles fully embedded in a background homogeneous environment with permittivity $\varepsilon_{\rm B} = 3.3$. The simulated EELS spectra have been convoluted with a Lorentzian function with FWHM of 0.15 eV and normalized to unity area. The impact parameter is chosen to be 0.1 nm outside the particle, i.e., $b - R = 0.1$ nm. The results from the EELS measurements are also shown (same as in Figure 5 of main text but in a reduced radius range). (**b**) Same as (**a**) but including the effects of nonlocal response through the GNOR model.

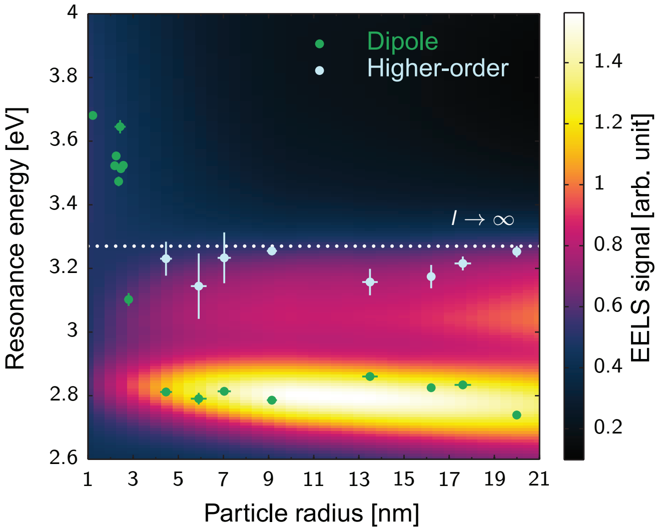

Supplementary Figure 11: EELS map with impact parameter outside the particles. Color plot of simulated EELS spectra convoluted with a Lorentzian function with FWHM of 0.15 eV and normalized to unit area (for each particle radius), as a function of energy and radius. The impact parameter is chosen to be 1 nm outside the particle, i.e., $b - R = 1$ nm.

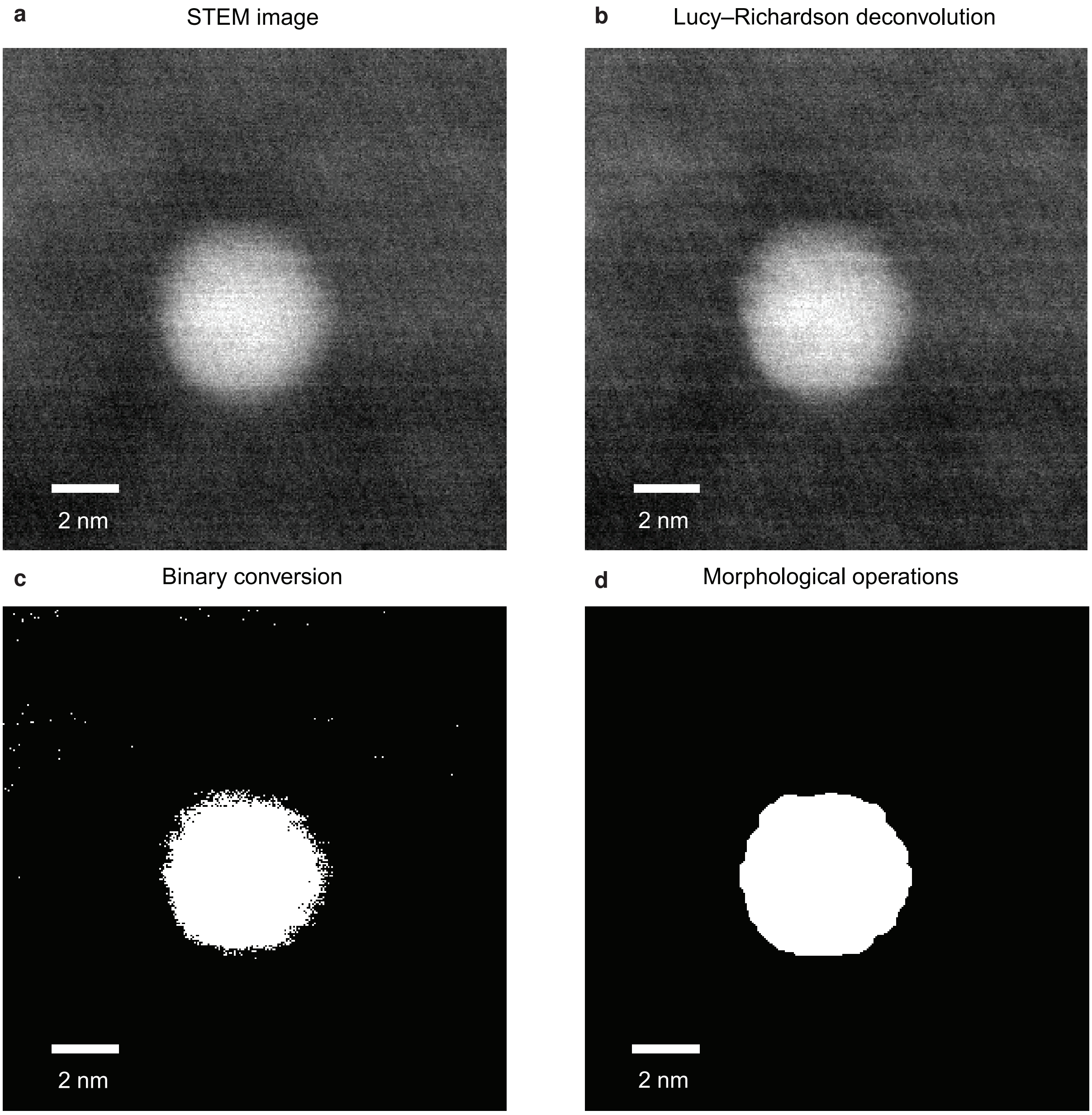

Supplementary Figure 12: STEM image analysis. (**a**) STEM image of silver nanoparticle with radius $R = 2.56 \pm 0.02$ nm. (**b**) STEM image sharpened using Lucy–Richardson deconvolution. (**c**) Grayscale-to-binary conversion. Grayscale threshold determined using Otsu's method. (**d**) Binary image after performing the following morphological operations: despeckle, fill, erode, and dilate. Scale bars are 2 nm.

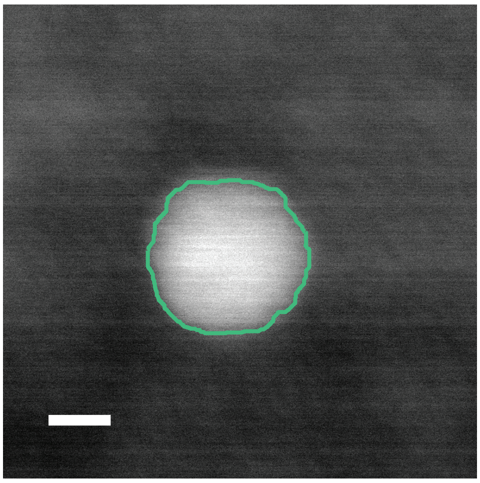

Supplementary Figure 13: STEM image analysis. Overlay of the boundary (green line) of the binary particle in Supplementary Figure 12(d) with the original STEM image. Scale bar is 2 nm.

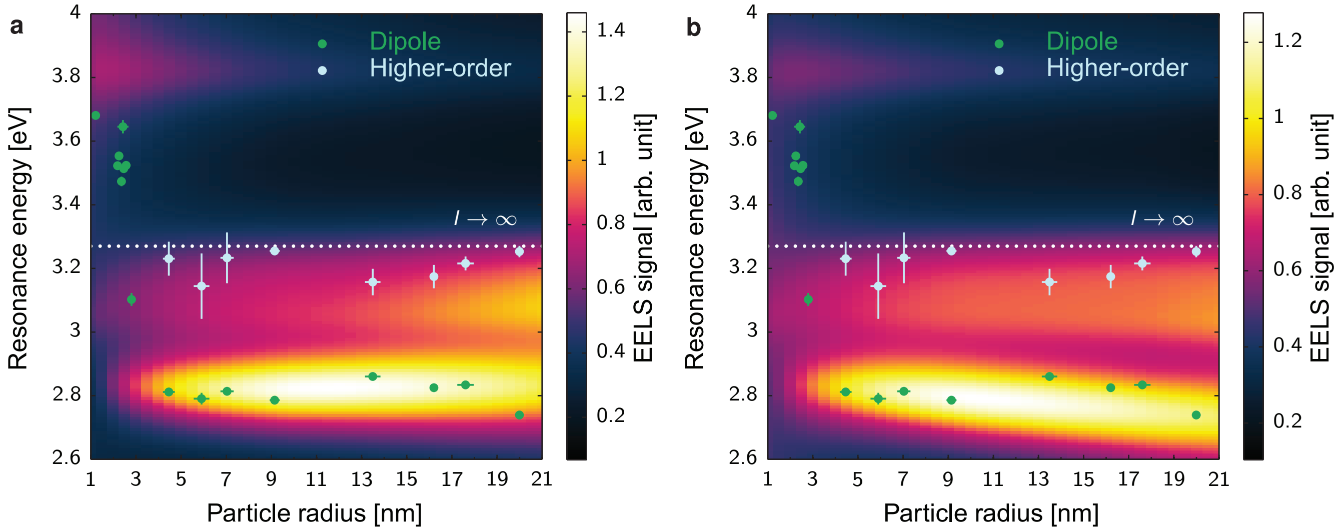

Supplementary Figure 14: EELS map with different thickness of the silicon nitride layers.

Same as Figure 5 in the main manuscript but with (**a**) 20% smaller silicon nitride thickness ($h_{\rm B} = 16$ nm, $h_{\rm T} = 12$ nm) and (**b**) 20% larger silicon nitride thickness ($h_{\rm B} = 24$ nm, $h_{\rm T} = 18$ nm). The impact parameter is $b - R = -1$ nm.

Supplementary Note 1: Theoretical models for EELS simulations

In this Note we describe in detail the different theoretical simulations, which we have performed to supplement and interpret the EELS measurements on SiN_x-encapsulated Ag nanoparticles. The EELS simulations shown in the main text are performed using the MNPBEM toolbox for MATLAB, which solves Maxwell's equations using the boundary-element method (BEM)[3] via the four-potential $(\phi, \mathbf{A})$.[4] An illustration of the radially-symmetric domain used for the simulations is shown in Supplementary Figure 5. A spherical Ag particle of radius R is conformally coated in a silicon nitride membrane. The coating geometry is modelled as the union of a circular membrane (i.e., disk) with diameter d_{mem} and height $h_{\text{B}} + h_{\text{T}}$, and a hemispherical cap with radius $R + h_{\text{T}}$ centered at the nanoparticle center . Here, h_{B} and h_{T} denote the thickness of the bottom and top layers, respectively. In the region denoted by the lengths l_{S} in Supplementary Figure 5, where the hemispherical cap connects to the circular membrane, a smoothing profile $r_{\text{S}}(z)$ is applied, given by the equation

$$r_{\text{S}}(z) = \pm\big[\sqrt{(R+h_{\text{T}})^2-(R-z)^2} + (l_{\text{S}}+h_{\text{T}}-z)^4/l_{\text{S}}^3\big] \quad \text{for} \quad h_{\text{T}} \leq z \leq h_{\text{T}} + l_{\text{S}}, \qquad (1)$$

where $z = 0$ is depicted by the horizontal dashed line in Supplementary Figure 5. The smoothing profile given by Eq. (1) ensures that there are no unphysical sharp domain boundaries. For the EELS simulations presented in the main text, we use $h_{\text{B}} = 20$ nm (5 nm from the TEM membrane and 15 nm from the additional silicon nitride deposition) and $h_{\text{T}} = 15$ nm. The smoothing length is chosen as $l_{\text{S}} = \min(R, 10\ \text{nm})$, such that the largest particles ($R > 10$ nm) show a fixed smoothing profile while the smoothing profile for the smallest particles ($R < 10$ nm) is size-dependent. For the truncation of the circular membrane (and thereby the simulation domain) we use $d_{\text{mem}} =$

$15 \times \max(R, h_{\mathrm{T}})$, corresponding to $d_{\mathrm{mem}} = 225$ nm for radii below 15 nm, and up to $d_{\mathrm{mem}} = 300$ nm for $R = 20$ nm. These truncation diameters are well-converged in the sense that the size of the simulation domain does not influence the calculated EELS signal. Finally, the dielectric function for silver is taken from tabulated data,[5] while for silicon nitride we use $\varepsilon_{\mathrm{SiN}_x} = 3.2$, which, as discussed in the main text, provides the best correspondence to the experimentally-measured dipole resonance energies.

To supplement the EELS simulations in the main text, we use our BEM model to also perform EELS simulations with the impact parameter positioned 1 nm outside the particle (i.e., $b - R =$ 1 nm), see Supplementary Figure 11. There are two main differences compared to the EELS simulations with the electron beam positioned 1 nm inside the particle (as shown in Figure 5 in the main text). First, the bulk plasmon is no longer excited by the electron beam. The second feature is the significantly stronger EELS signal from the dipole mode compared to that of the HO modes. We observe both of these features in our EELS measurements, which is why we determine the resonance energy of the dipole modes from EELS data acquired a few nanometers outside the particle surface (see also the discussion in relation to Figure 3).

We have also examined the influence of the thickness of the silicon nitride encapsulating layer using our BEM model. In Supplementary Figure 14, we show EELS simulations (with impact parameter $b - R = -1$ nm) where we vary the thickness of the silicon nitride layer by $\pm 20\%$. As in Figure 5 of the main manuscript, we superimpose the experimentally-measured resonance energies of the dipole and HO modes. The simulated resonance energies of both the dipole and HO modes show no significant change for $R \lesssim 10$ nm, while for $R \gtrsim 10$ nm a small blueshift (redshift) of

the plasmon modes for thinner (thicker) silicon nitride is observed compared to Figure 5 of the main manuscript. Importantly, these results demonstrate that our experimental observations of the dipole and HO modes are robust with respect to small variations of the thickness of the silicon nitride layers.

Fully-embedded approximation and nonlocal response As nonlocal response is not incorporated in the MNPBEM Toolbox, we assess the effects of nonlocality by using the analytical EELS model of Refs. 6 and 7, in which the system of a spherical metal particle *fully embedded* in a homogeneous dielectric medium is solved. However, we must first consider whether the fully-embedded approximation is a valid scenario for the EELS measurements of the smallest nanoparticles, where non-classical features are observed.

In relation to the classically maximal allowed energy of the HO modes, given by the $l \to \infty$ limit of Eq. (1) in the main text, we estimated the effective background permittivity (in a fully embedded quasistatic scenario) as $\varepsilon_{\mathrm{B}} = 3.3$, a value very close to $\varepsilon_{\mathrm{SiN}_x} = 3.2$ used in our BEM simulations to model the encapsulating silicon nitride layers. The small difference between these two values provides support for the interpretation that the SP modes of the smallest particles ($R < 10$ nm) behave as in a fully embedded homogeneous background environment. To quantitatively substantiate this interpretation, we perform EELS simulations of spherical silver particles embedded in a homogeneous background environment with permittivity $\varepsilon_{\mathrm{B}} = 3.3$. For this simpler geometry, the EELS signal can be calculated using an analytical approach, detailed in Refs. 6–8, as long as the impact parameter is outside the sphere ($b > R$), the background permittivity is purely real-valued, and, finally, the electron velocity is smaller than the speed of light in the background medium

($v < c/\sqrt{\varepsilon_B}$) to avoid effects of Cherenkov radiation. Fortunately, the two first conditions are suitable for our case, while the third condition requires us to use a slightly lower acceleration voltage ($U = 100$ kV) in our analytical calculation than in the experimental setup ($U_{exp} = 120$ kV). However, we have checked that this slight decrease in electron velocity (from $v = 0.59c$ to $v = 0.55c$) does not influence the EELS signal notably by comparing the analytical approach to numerical BEM simulations of fully-embedded spherical nanoparticles.

The analytical EELS calculation of fully-embedded silver particles is shown in Supplementary Figure 10(a) for impact parameters only 0.1 nm from the particle surface ($b - R = 0.1$ nm). This value for the impact parameter is chosen to maximize the signal from the HO modes. We find that the fully-embedded approximation captures quite well our experimental observations for $R < 10$ nm, providing strong support for the interpretation that the SP modes of the encapsulated silver particles behave as in a homogeneous background environment for these particle radii. However, as with the BEM simulations of the encapsulated conformal geometry, we find that these calculations based on classical electrodynamics do not agree with our experimental observations for $R < 4$ nm, where the HO modes disappear and the dipole mode shows a rapid increase in resonance energy with decreasing particle size.

To assess the origin of these two non-classical observed effects, i.e., the disappearance of the HO modes and the blueshift of the dipole mode, we also perform EELS calculations of fully-embedded particles taking into account nonlocal response in the silver nanoparticles.[9,10] As shown in Refs. 6 and 7, the analytical approach used for the classical calculations can be extended to include nonlocal response in the metal nanoparticle. Nonlocality in the metal is taken into account

through the generalized nonlocal optical response (GNOR) model,[9–11] which extends the nonlocal hydrodynamic model[12] by including the important effects of electron diffusion. The GNOR model describes size-dependent resonance shifts and linewidth broadening of localized surface plasmon excitations in noble metal particles.[9] These effects have been shown to be stronger for increasing multipolar order l.[10] For the GNOR calculations we use the following parameters appropriate for silver[11]: $\hbar\omega_{\rm p} = 8.99$ eV, $\hbar\gamma = 0.025$ eV, $v_{\rm F} = 1.39 \times 10^6$ m/s, and $D = 3.61 \times 10^{-4}$ m^2/s. Here, $\omega_{\rm p}$ is the plasma frequency, γ is the Drude damping rate, $v_{\rm F}$ is the Fermi velocity, and D is the diffusion constant. The core-response, i.e., $\varepsilon_{\rm core}(\omega)$, is determined from experimental data[5] by the subtraction of the Drude part $-\omega_{\rm p}^2/(\omega^2 + i\gamma\omega)$.

The EELS calculation of fully-embedded silver nanoparticles described by the GNOR model is shown in Supplementary Figure 10(b), along with the EELS measurements. The strongest EELS signal stems from the dipole mode, while the weaker higher-energy signal is from the excitation of the HO modes. The GNOR calculations show several important differences compared to the classical calculation in Supplementary Figure 10(a). First of all, in the GNOR model the HO modes are not bound by the classical $l \to \infty$ energy limit, thereby extending beyond the energy $\hbar\omega_{l\to\infty} = 3.27$ eV and providing better agreement with the measured HO resonance energies. The second feature is the decrease in strength of the HO modes with decreasing particle radii. This is a consequence of the size- and l-dependent damping of localized surface plasmons, which is a main feature of the GNOR model. In particular, with decreasing particle radius and increasing multipolar order l the linewidth of the surface plasmon increases.[10] From Supplementary Figure 10(b) we see that the calculated EELS signal from the HO modes decreases with decreasing particle radius, and almost completely disappears for particle radius below approximately 4 nm. Importantly, we

also observe this effect in our EELS measurements, where EELS spectra of particles with radii below 4 nm only show a single SP peak [see for instance Figure 4(h) in the main text]. From this agreement, we find strong support for the interpretation that the disappearance of the HO modes for very small particle radii is a consequence of nonlocal response, i.e., a fundamental property of the free-electron gas in silver nanoparticles, and not due to, e.g., lack of instrumental resolution. The third and final feature in the GNOR calculations is the increase in resonance energy of the dipole mode as the particle radius decreases. We find that the GNOR calculations are only in qualitative agreement with the EELS measurements, since the experimentally measured blueshift is much larger than predicted by theory. This discrepancy suggests that other effects in silver besides nonlocal response play a part in the observed blueshift of the dipole mode.

Extinction cross section To provide context for the use of EELS to excite HO modes, we present in this subsection calculations of fully-embedded silver nanoparticles using a plane wave as an excitation source (instead of a swift electron beam). Plane wave excitation is typical in far-field measurements. In Supplementary Figure 1, we show the cross-sectional efficiency (extinction cross section normalized to sphere cross section) of a silver nanoparticle embedded in a homogeneous environment with permittivity $\varepsilon_{\mathrm{B}} = 3.3$. The calculations are performed using Mie theory.[13] For the nanoscopic particles under consideration, we find only a single resonance, associated with the excitation of the dipole mode. The resonance energy decreases with increasing particle size due to retardation. We point out that no higher-order modes are observed, since the excitation source is a plane wave which couples inefficiently to modes of higher angular momentum.

Supplementary Note 2: Conformal silicon nitride coating of silver nanoparticles

We have thoroughly investigated the morphology of the silicon nitride coating to ensure that the nanoparticles are conformally coated. In this Note, we present two different experimental analyses confirming the conformal morphology of the silicon nitride layer. For the first analysis, we prepare a cut-out of a representative TEM sample using focused ion beam (FIB) milling for production of a lamella, used to obtain a cross-sectional view of the sample. The sample used for this purpose has a thicker silicon nitride encapsulating layer (approximately 33 nm below and 27 nm above the particles) compared to the sample used in the main text (see Methods section in main text). The additional thickness in the bottom layer stems from the approximately 5 nm thick silicon nitride TEM membrane. In order to protect the region of the sample we wanted to investigate, we deposited in situ a layer of platinum. Afterwards, the TEM lamella was cut free on three sides and at the bottom (in the Si substrate under the silicon nitride layer) before welding the lamella to an etched tungsten tip using in situ platinum deposition. The lamella was detached from the sample using the FIB and welded on a TEM grid using the gas injection system. Finally the lamella was carefully thinned to approximately 80 nm with the FIB to allow investigation in the TEM. In Supplementary Figure 2(a) we show a cross-sectional view of our FIB-prepared lamella. The contrast in STEM images depends on both the thickness and atomic number of the materials in the sample. As the thickness of the lamella is constant in the viewing direction ($\sim$ 80 nm), the contrast seen in Supplementary Figure 2(a) originates to a good approximation only from differences in atomic number. In particular, the top platinum layer from the FIB procedure is visible along with the silver nanoparticles encapsulated in the silicon nitride coating. It is evident that the silicon nitride layer conformally coats the nanoparticles with larger domes in the surface

topography around larger particles. The image contrast in the dome region is due to both the encapsulating nitride layer and the grainy platinum layer; the latter in particular is responsible for the non-uniform appearance of the domes. We stress, however, that the silicon nitride domes are uniform, as is also seen in Supplementary Figure 2(b), which displays a scanning electron microscope (SEM) top-view image of the sample before the FIB procedure. The SEM image shows clear uniform domes in the silicon nitride and, additionally, provides further evidence for the conformal morphology of the encapsulating layer.

In the second experimental analysis, we use EELS and energy-dispersive X-ray spectroscopy (EDS) measurements to study the composition of our sample. These measurements are acquired on the same sample as the one studied in the main text. In Supplementary Figure 3(b-c) we show EDS and EELS spectra, respectively, acquired at the two positions shown in Supplementary Figure 3(a). One of the impact positions is close to a silver nanoparticle of radius $R = 22.1 \pm 0.7$ nm (shown in blue), while the other position is at a distance from the particle (shown in red). On comparing the EDS spectra from these two positions [Supplementary Figure 3(b)], we find similar EDS signal from the two positions with a clear peak from silicon. Due to the low atomic number, the EDS signal from nitrogen is quite weak and not clearly distinguished. However, elemental quantification from the EELS spectra (which is more suitable for materials with low atomic number) shows the same silicon-to-nitrogen ratio at the two positions, indicating that the two regions are compositionally the same. Elemental quantification from the EDS spectra also shows that the composition of the coating close to and far away from the particle is similar. Additionally, the EELS spectra in Supplementary Figure 3(c) reveal that the thickness of the coating is larger in the vicinity of the particle, which explains the halo around the nanoparticle in Supplementary Figure 3(a).

Combining the information from the correlated EDS and EELS measurements, we conclude that the coating composition does not change in the vicinity of the particle while the coating thickness increases, providing further evidence for the conformal morphology of the coating.

Supplementary Note 3: Morphology of silver nanoparticles

To assess the effect of encapsulation on the morphology of the silver nanoparticles, we prepared an additional sample without the top layer of silicon nitride (i.e., bare silver nanoparticles deposited on a 20 nm thick silicon nitride substrate). In Supplementary Figure 4 we display STEM micrographs of selected nanoparticles from the two different samples (with and without the silicon nitride encapsulation). In both cases the nanoparticles appear almost spherical in shape. However, we observe that the non-encapsulated nanoparticles [Supplementary Figure 4(a-b)] show a smooth morphology, while the encapsulated nanoparticles show a faceted icosahedral morphology [Supplementary Figure 4(c-d)]. This difference in morphology is attributed to oxidation and formation of silver sulfide on the non-encapsulated nanoparticles that cannot be avoided without the protective top layer. This observation evidences that the encapsulation of the silver nanoparticles with a top layer of silicon nitride enables us to obtain high-quality pure silver nanoparticles with well-defined icosahedral morphologies.

Supplementary Note 4: Dielectric constant of encapsulating silicon nitride

Using EELS data acquired by positioning the focused electron beam in a particle-free region, we can estimate the dielectric constant of the encapsulating silicon nitride. We use the Kramers–Kronig method[1] to extract the real and imaginary parts of the dielectric constant. This method requires EELS data with a large energy range, so we acquire energy losses separated by 0.25 eV, giving us a total energy range of 512 eV (i.e., dispersion of 0.25 eV pr. channel). The EELS-extracted dielectric constant is displayed in Supplementary Figure 6 using two methods to remove the zero-loss peak (part of the Kramers–Kronig procedure). The red lines (blue lines) show the result using the reflected-tail method (fitting of sum of Gaussian and Lorentzian squared functions) to remove the zero-loss peak. The black lines show the previous result for bulk silicon nitride with chemical composition Si_3N_4.[2] While providing qualitative insight, we also find a strong variation in the extracted dielectric constant depending on the zero-loss peak removal technique, which prevents more accurate insight to the exact values for the dielectric constant of our encapsulating silicon nitride layer. We have therefore decided to use a pragmatic approach to determining the value of $\varepsilon_{\mathrm{SiN}_x}$, where, as described in the main text, we ensure the best correspondence between the simulated and measured dipole resonance energies.

Supplementary Note 5: Spatial dependence of the weight of higher-order modes relative to the dipole mode

We here provide a brief discussion of the b-dependence of the excitation weights of HO modes vis-à-vis the dipole mode. Additionally, we comment on its relation to the field-localization of the plasmons and the exciting electron beam.

From a basic perspective, we note that the extents of the plasmon and electron beam fields are of comparable magnitudes. Specifically, the field of an l-order multipolar plasmon of an isolated sphere decays approximately as $\sim r^{-(l+2)}$ (quasistatic limit) and exhibits a $1/e$ decay length $\sim R/e^{(l+2)}$, significantly shorter than the particle radius, and decreasing exponentially with multipole order. The radial extent of the field associated with the electron beam is $\sim v\gamma_v/\omega$ (with Lorentz factor γ_v), on the order of 50 nm in our measurements; but is sharply peaked (in fact divergently so, in the limit of vanishing beam width) about its origin. Evidently, appreciable electron energy loss requires significant overlap of the fields of both plasmon and probe: the tighter confinement of HO modes restricts their excitation to grazing impacts. Similarly, the increasingly inhomogeneous nature of the HO modes require a comparably inhomogeneous excitation field for significant overlap – otherwise the overlap is zeroed by repeated oscillations – thus compounding the severity of the previous statement.

A fully quantitative explanation originates in the quasistatic multipolar expansion of the electron energy loss for a single sphere (in vacuum, with impact $b > R$)[6,8,14]

$$\Gamma(\omega) = \alpha_v \frac{4R}{\pi v} \sum_{l=1}^{\infty} \sum_{m=-l}^{l} \left(\frac{\omega R}{v}\right)^{2l} K_m^2\left(\frac{\omega b}{v}\right) \mathrm{Im}\left[\frac{l\varepsilon(\omega) - l}{l\varepsilon(\omega) + l + 1}\right] \frac{1}{(l+m)!(l-m)!}, \quad (2)$$

with $\alpha_v \equiv e^2/4\pi\varepsilon_0\hbar v$. Though still an expansive formula, it is clear that the impact parameter b enters only the Coulomb term $K_m^2(\omega b/v)$; with the exception of the $m = 0$ contribution, this term diverges as $K_m^2(x) \sim [2^{|m|-1}(|m|-1)!x^{-|m|}]^2$ for $x \ll 1$. For sufficiently small $\omega b/v$ the $m = \pm l$ terms then dominate all other m, allowing the approximate form

$$\Gamma(\omega) \simeq \alpha_v \frac{2R}{\pi v} \sum_{l=1}^{\infty} n_l \left(\frac{R}{b}\right)^{2l} \mathrm{Im}\left[\frac{l\varepsilon(\omega) - l}{l\varepsilon(\omega) + l + 1}\right], \tag{3}$$

with coefficients $n_l \equiv 2^{2l}[(l-1)!]^2/(2l)!$. From this simpler formula, it is clear that the weight of moderately high l-order modes is enhanced as R/b approaches unity from below, i.e., as b is reduced towards R (while very high angular momenta remain suppressed owing to the coefficients n_l which approach $n_l \sim \sqrt{\pi}/l^{3/2}$ for large l).

Supplementary Note 6: Image analysis

The analysis of the STEM images is performed in MATLAB using the Image Processing Toolbox. Each image contains only a single particle, see Supplementary Figure 12(a) for an example. The image is first sharpened using the built-in Lucy–Richardson deconvolution scheme in MATLAB [Supplementary Figure 12(b)]. For the deconvolution, the spatial distribution of the STEM probe is assumed to be Gaussian with a full-width at half-maximum of 5 Å. The number of iterations in the deconvolution is usually set to 3 to avoid deconvolution-induced artifacts. The sharpened grayscale image is then converted to a binary image [Supplementary Figure 12(c)]. The grayscale threshold limit for the conversion is determined using Otsu's method, which is included in MATLAB. Afterwards, we perform a number of morphological operations on the binary image to clearly determine the boundary of the particle. In particular, we remove isolated white pixels, fill isolated interior black pixels, and finally, erode and dilate the binary image with a disk-shaped structuring element [Supplementary Figure 12(d)]. The boundary of the white binary particle is then extracted [Supplementary Figure 13] and fitted to a circle and ellipse. Assuming the particle to be spherical in shape, we take the radius of the fitted circle R as the particle radius. The difference between the major axis a and minor axis b of the fitted ellipse is used to determine the error $\Delta R = a - b$ in the particle radius.

Supplementary References

1. Egerton, R. F. *Electron Energy Loss Spectroscopy in the Electron Microscope* (New York: Springer, 2011), 3rd edn.

2. Philipp, H. R. Optical properties of silicon nitride. *J. Electrochem. Soc.* **120**, 295–300 (1973).

3. Hohenester, U. & Trügler, A. MNPBEM–A Matlab toolbox for the simulation of plasmonic nanoparticles. *Comp. Phys. Commun.* **183**, 370–381 (2012).

4. García de Abajo, F. J. & Howie, A. Retarded field calculation of electron energy loss in inhomogeneous dielectrics. *Phys. Rev. B* **65**, 115418 (2002).

5. Johnson, P. B. & Christy, R. W. Optical constants of the noble metals. *Phys. Rev. B* **6**, 4370–4379 (1972).

6. García de Abajo, F. J. Optical excitations in electron microscopy. *Rev. Mod. Phys.* **82**, 209–275 (2010).

7. Christensen, T. *et al.* Nonlocal response of metallic nanospheres probed by light, electrons, and atoms. *ACS Nano* **8**, 1745–1758 (2014).

8. García de Abajo, F. J. Relativistic energy loss and induced photon emission in the interaction of a dielectric sphere with an external electron beam. *Phys. Rev. B* **59**, 3095–3107 (1999).

9. Mortensen, N. A., Raza, S., Wubs, M., Søndergaard, T. & Bozhevolnyi, S. I. A generalized nonlocal optical response theory for plasmonic nanostructures. *Nat. Commun.* **5**, 3809 (2014).

10. Raza, S., Bozhevolnyi, S. I., Wubs, M. & Mortensen, N. A. Nonlocal optical response in metallic nanostructures. *J. Phys. Cond. Matter.* **27**, 183204 (2015).

11. Raza, S., Wubs, M., Bozhevolnyi, S. I. & Mortensen, N. A. Nonlocal study of ultimate plasmon hybridization. *Opt. Lett.* **40**, 839–842 (2015).

12. Boardman, A. *Electromagnetic surface modes. Hydrodynamic theory of plasmon-polaritons on plane surfaces.* (John Wiley and Sons, Chichester, 1982).

13. Bohren, C. F. & Huffman, D. R. *Absorption and Scattering of Light by Small Particles* (John Wiley & Sons, Inc., New York, 1983).

14. Ferrell, T. L., Warmack, R. J., Anderson, V. E. & Echenique, P. M. Analytical calculation of stopping power for isolated small spheres. *Phys. Rev. B* **35**, 7365–7371 (1987).
